# Systematic Review on the Efficacy and Safety of Herbal Medicines for Vascular Dementia

**DOI:** 10.1155/2012/426215

**Published:** 2011-10-20

**Authors:** Sui Cheung Man, Kam Wa Chan, Jia-Hong Lu, Siva Sundara Kumar Durairajan, Liang-Feng Liu, Min Li

**Affiliations:** School of Chinese Medicine, Hong Kong Baptist University, Kowloon Tong, Hong Kong

## Abstract

We present a systematic review of existing research that aims to assess the efficacy and safety of herbal medications (HM), as either monotherapy or adjunct to orthodox medications (OM), mainly comprised of cholinesterase inhibitors, for vascular dementia (VaD). We included 47 studies conducted in mainland China, each testing different HM. Of 43 HM monotherapy studies, 37 reported HM to be significantly better than OM or placebo; six reported similar efficacy between HM and OM. All four HM adjuvant studies reported significant efficacy. No major adverse events for HM were reported. Heterogeneity in diagnostic criteria, interventions and outcome measures hindered comprehensive data analysis. Studies suggested that HM can be a safe and effective treatment for VaD, either alone or in conjunction with OM. However, methodological flaws in the design of the studies limited the extent to which the results could be interpreted. Thirty most commonly used herbal constituents, including Rhizoma Chuanxiong (Chuanxiong in Chinese), Radix Polygoni Multiflori (Heshouwu in Chinese) and Radix Astragali (Huangqi in Chinese). were ranked. Further multi-center trials with large sample sizes, high methodological quality and standardized HM ingredients are necessary for clinical recommendations to be made.

## 1. Introduction

Vascular dementia (VaD) is one of the most common forms of dementia after Alzheimer's disease (AD) [1], and the most frequent cause of dementia in the elderly [2]. First described as arteriosclerotic dementia [3], VaD is defined as loss of cognitive function resulting from ischemic, hemorrhagic brain lesions (such as border zone infarcts and ischemic periventricular leukoencephalopathy) or hypoperfusion, due to cerebrovascular disease or cardiovascular pathology [4]. Incomplete microangiopathic infarcts due to fibrohyalinosis are regarded as the major pathophysiological manifestation [5] of VaD. While AD is characterized by memory impairment, VaD is characterized by executive dysfunction [6] and behavioral psychological symptoms such as apathy, abulia, opposition, agnosia [7], anxiety [8], depression [9], and suicidal thoughts [9]. Cognitive impairment is relatively mild as compared to AD. 

VaD accounts for approximately 30% of dementia in the world today [10]. In Europe, out of 3.7 million of people with clinical dementia, 800,000 have a diagnosis of VaD [11]. The prevalence rate of VaD is around 1–4% in Western developed nations [12]. Recently, in mainland China, a nationwide investigation found the prevalence of VaD to be around 0.8% [13]. The total annual cost (direct, illness related and cost arise from informal care) of dementia in developing countries is estimated to be at least USD 73 billion [10]. A study in Denmark revealed the annual cost per demented person to be DKK 77,000 (approximately USD 14,114) [14]. Thus, the total healthcare cost for VaD patients is highest among all other forms of dementia [15], and the frequency of VaD is increasing exponentially for people over the age of 65 years old [16]. If current trends continue, VaD will become an increasingly significant public health problem in the 21st century.

Drugs currently used in the treatment of VaD include cholinesterase inhibitors (donepezil, rivastigmine, and galantamine) [17] and non-cholinergics (memantine, nimodipine, hydergine, nicergoline, CDP-choline, folic acid [18], posatirelin, propentofylline, and pentoxifylline [19]). These orthodox medications (OM) have some efficacy [20]. Preventive therapeutic strategies aiming at reducing cerebrovascular risk factors [17] are also considered by patients likely to develop VaD. As yet, there is no compelling evidence that any of these strategies are effective, and no single intervention can be recommended for the prevention of VaD [21]. This creates a difficult and frustrating situation for sufferers of the disease, their caregivers, and healthcare providers [18], as well as for healthy people hoping to avoid developing VaD.

Owing to the limitations of OM and therapeutic prevention, some patients resort to herbal medications (HM). Traditionally, a number of herbs have been used for cognitive disorders. For example, *Artemisia absinthium* (Wormwood) was used in traditional European medicine to restore cognitive functions [22]. *Melissa officinalis* (Lemon balm), also widely used in Europe, has been claimed to restore memory [23]. Since the 16th century, Europe, *Salvia lavandulaefolia* (Spanish sage) and *Salvia officinalis* (common sage) have been reported as being effective for improving memory [22]. *Bacopa monniera* (water hyssop) has been used in Ayurvedic medicine to improve memory and intellectual functions. *Centella asiatica* (Asiatic pennywort), another Ayurvedic remedy, when combined with milk, is also given to improve memory [24]. *Withania somnifera* root is classed among the rejuvenative tonics in Ayurvedic medicine and is known to sharpen memory [25]. *Codonopsis pilosula* root (Dangshen in Chinese), *Biota orientalis* leaves (Cebaiye in Chinese), and *Polygala tenuifolia* root (Yuanzhi in Chinese) have been used in traditional Chinese medicine (TCM) for amnesia [26, 27]. Some compounds with cognition-improving properties have been isolated from various plants. EGb 761, an extract from the leaves of the tree *Ginkgo biloba*, originally used in Western medicine for circulatory disorders [28], shows reversal of decline in cognitive function and of cerebral insufficiency in numerous studies [29], and is now mainly used in VaD as well [30]. In another study, hyperforin, isolated from *Hypericum perforatum*, a herb used in Portuguese folk medicine, appears to enhance cognitive function [31]. 

In China, a nation with its own system of medicine that has been continuously documented over two thousand years, the incorporation of Chinese herbal medicine (CHM) with Western medicine in the treatment of dementia has become a standard in recent decades. *Salvia miltiorrhiza* Bge. (Danshen in Chinese) and *Pueraria thomsonii* Benth. (Gegen in Chinese), commonly used herbs in the Chinese materia medica for the treatment of cardiocerebrovascular symptoms, are well tolerated and effective in improving vascular function and structure. Thus, either one might be able to effectively intervene in the pathophysiological cascade of VaD [32]. An animal study revealed that glossy privet fruit (*Ligustrum lucidum* Ait.), a kidney-tonifying Chinese herbal medicine, inhibits neural cell apoptosis following the onset of vascular dementia by reducing apoptotic signals induced by cerebral ischemia/hypoxia [33]. A number of proprietary herbal medicines may also be effective for VaD. According to a study, Chunghyul-dan, which possesses therapeutic effects for microangiopathy, could be useful to inhibit the development of VaD [34]. Huperzine A (HupA), a cholinesterase inhibitor naturally derived from the Chinese herb *Lycopodium serratum* or *Huperzia serrata*, has even better penetration through the blood-brain barrier, higher oral bioavailability, and longer duration of AChE inhibitory action than tacrine, donepezil, and rivastigmine [35]. Its anticholinesterase activity is stronger than galantamine (a commonly used drug to treat Alzheimer's disease and various memory impairments) [26]. Its ability to improve memory deficits in elderly people with VaD and AD has been demonstrated, with minimal peripheral cholinergic side effects and no unexpected toxicity [35]. These reports suggest that comprehensive investigation of the efficacy and safety of HM is worthwhile; the results could lead to better treatment of VaD as well as effective prevention.

In a previously conducted systematic review [36], we looked for clinical trials for Alzheimer's disease (AD), and found an even larger number of clinical trials conducted on VaD patients, mainly from mainland China. Because a large proportion of patients have both VaD and AD pathologies [37], and because the current OM treatments for both types of dementia are similar, we have systematically reviewed the clinical trials of HM conducted on VaD patients in this study.

## 2. Objective

This systematic review was conducted to assess the safety and efficacy of HM, as either monotherapy or adjunct to OM in the treatment of VaD.

## 3. Method

### 3.1. Inclusion Criteria

All published studies reporting randomized, controlled clinical trials comparing HM as monotherapy or adjuvant therapy, with placebo or OM as controls, were included. No restriction on the language of publication was imposed. As there is a lack of a single, specific criterion for the diagnosis of VaD [38], we accepted the use of the following instruments: Diagnostic and Statistical Manual of Mental Disorders (DSM-III, DSM-III-R [39], and DSM-IV [40]), the International Classification of Diseases, 10th Revision (ICD-10) [41]; the State of California Alzheimer's Disease Diagnostic and Treatment Centers (ADDTC) scale [42]; the Hachinski Ischemic Scale (HIS) [43]; the National Institute of Neurological Disorders and Stroke-Association Internationale pour la Recherche et l'Enseignement en Neurosciences scale (NINDS-AIREN) [44]. Trials with participants possessing other forms of dementia (Alzheimer's disease, Lewy body dementia, and frontotemporal dementia) were excluded. There was no restrictions on the ethnicity, gender, age, or disease duration of the participants in the trials. The HM interventions could be either (1) a single herb, (2) a preparation containing multiple herbs, (3) extracts from an herb, or (4) proprietary herbal products. They had to have been used alone or coadministered with conventional medications (OM). The control intervention had to have been either (1) a placebo, or (2) OM. Trials of all durations were included. Crossover studies were also accepted if the first phase fulfilled the above criteria. Outcome measures of interest were (1) Mini-Mental Status Examination (MMSE) [45], (2) Activities of Daily Living Scale (ADL) [46], and (3) Hasegawa Dementia Scale (HDS) [47]. The safety profile might be represented in (5) adverse effect count, (6) biochemical indications, or (7) number of withdrawals due to adverse events.

### 3.2. Search Strategy and Method of Review

We identified trials from the following electronic databases: (1) Ovid MEDLINE In-Process and Other Non-Indexed Citations and Ovid MEDLINE; (2) CINAHL; (3) EMBASE; (4) EBM Reviews; (5) AMED; (6) ACP Journal Club; (7) Cochrane Central Register of Controlled Trials; (8) Cochrane Database of Systematic Reviews; (9) Cochrane Methodology Register; (10) Database of Abstracts of Reviews of Effects; (11) Health Technology Assessment; (12) National Health Service Economic Evaluation; (13) China National Knowledge Infrastructure (CNKI); (14) Chinese Sci and Tech Journals (VIP); (15) CBM disc; (16) China Doctor Dissertations Full-Text Database; (17) China Master Theses Full-text Database. The search conducted in March 2011 followed a strategy (Table 1) developed with reference to a Cochrane review on herbal medicine [48], regardless of language and publication status. Hand-search of a list of Chinese and English journals was carried out to find the latest studies. We also referred to the reference lists of relevant papers to identify potential studies.

Two independent reviewers (K. W. Chan and S. C. Man) assessed the trials for their eligibility. The inclusion of trials was confirmed upon consensus of reviewers. Risk of bias assessment of the trials was performed according to the revised Consolidated Standards of Reporting Trials (CONSORT) statement [49]. Any disagreement was settled by discussion.

## 4. Results

### 4.1. Description of Included Studies

Using the search strategy as described, 116 studies were identified. Upon full-text examination we excluded 69, on the basis that (1) 13 were not randomized controlled trials, (2) four were repeat publications, (3) three did not state their inclusion criteria, (4) one included other forms of dementia, (5) 44 did not disclose adequate baseline information, and (6) four involved the use of non-HM intervention, such as acupuncture. These disqualifications left 47 studies for this systematic review.

There were a total of 3725 participants (2423 male, 1302 female) in the 47 included trials. Among them, three had cross-over design while the remaining were parallel design studies. The age of participants ranged from 45–89 years old, and their disease duration ranged from two months to 12 years. Thirty-two studies were performed in a single center; one was performed in multiple centers; 14 trials did not give this information. The duration of trials lasted from one to seven months. All of the trials were conducted in mainland China, and all the subjects were of Chinese ethnicity.

### 4.2. Risk of Bias (Table 2)

We adopted the checklist of items suggested by CONSORT [50] in the evaluation of methodological quality (risk of bias). The checklist was divided into five sections, namely, title and abstract, introduction, methods, results, and discussion.

Except for 4 studies [51–54], all had adequate information on the title and abstract. Four studies [55–58] did not give an appropriate introduction. 

With regard to method, none of the 47 studies reported details for sample size calculations. Eight [51–54, 57, 59–61] did not clearly state their objective of study. All but one study [62] poorly reported their randomization and blinding. Statistical method was not reported in 13 studies [51–53, 58, 59, 63–70]. 

For the results section, only two studies [62, 71] reported with a flowchart. Patient recruitment, outcomes and estimation, and ancillary analyses were mentioned by the majority of studies. More than half of the studies did not report adverse event count. 

In the discussion section, both the interpretation and overall evidence were adequately reported by the studies; generalizability, however, was not sufficiently illustrated by 13 studies.

### 4.3. Randomization (Table 2)

All of the included studies claimed to have allocated participants randomly to study groups. Six [72–77] reported the use of computer-generated sequences; the other 41 studies did not provide any description of how randomization was achieved.

### 4.4. Allocation Concealment (Table 2)

Except for one study [71] which clearly stated that it did not use any blinding methods, 32 studies did not report whether they applied blinding or not. For those studies which reported the use of allocation concealment, 6 were single blind [52, 57, 66, 78–81], and 8 were double blind [55, 56, 62, 76, 82–85].

### 4.5. Eligible Criteria (Table 3)

Only two studies [82, 86] used a single diagnostic criterion to select participants. Other studies used two or more diagnostic criteria. Among them DSM III and IV were the most commonly adopted (42 studies used it) [39, 40]. The other criteria commonly used, listed in descending order of frequency, were HIS [43] (27 studies), MMSE [45] (25 studies); HDS [47] (17 studies); ADL [46] (11 studies); clinical dementia rating scale (CDR) [87] (6 studies); NINDS-AIREN [44] (five studies); ICD-10 [41] (four studies). Other measurement scales used included the scale for the differentiation of syndromes of vascular dementia (SDSVD) [88], Functional Activities Questionnaire (FAQ) [89], and self-derived criteria (SELF). Furthermore, some studies also carried out diagnostic imaging such as CT and magnetic resonance imaging (MRI) for participant selection.

### 4.6. Baseline Characteristics and Outcome Measures (Table 3)

A number of batteries were employed to evaluate the baseline characteristics and outcome measures. The most commonly employed set of evaluative questionnaires included MMSE [45] (38 studies); HDS [47] (23 studies); ADL [46] (20 studies); Berg Balance Scale (BBS 8 studies); SELF (5 studies); memory quotient (MQ 1 study); geriatric dementia scale (GDS 1 study). Moreover, diagnostic imaging such as electroencephalography (EEG 6 studies) and CT (2 studies) were carried out. A number of studies also took into account the changes in hemodynamics (21 studies) and transcranial doppler (TCD 3 studies) as part of the outcome measures. Lastly, biochemical analysis, such as changes in the level of superoxidase dismutase (SOD), malondialdehyde (MDA), homocysteine (HCY), testerone (T), and 17 beta-estradiol (E2) were also adopted in assessing the efficacy and safety of interventions in the studies.

The different batteries used in the studies resulted in variation in outcome measures. As the data were not suitable for meta-analysis, only qualitative appraisal could be carried out.

### 4.7. Herbal Medicine as Monotherapy (Table 3)

There were altogether 43 trials testing herbal medicine as a monotherapy for VaD. Among them 15 studies compared different HM preparations with Piracetam alone (a nootropic agent). One study compared HM with another stronger nootropic compound Aniracetam. One study compared HM with Piracetam + hydergine and one study compare HM with Piracetam + Vitamin E + respiratory stimulant Duxil (Almitrine). Hydergine, (also known as ergoloid mesylates, another nootropic agent), was tested alone against HM in 11 studies. Five studies reported HM having similar efficacy to these nootropics; the remaining 23 claimed HM to be significantly better.

Seven studies compared HM with Duxil (Almitrine) alone, a respiratory stimulant originally used to treat patients with chronic obstructive pulmonary disease. In one study, HM is compared with Duxil + Nimodipine (a dihydropyridine calcium channel blocker for the treatment of high blood pressure). One study reported HM to have similar efficacy with Duxil; the other seven claimed that HM is better than Duxil.

Furthermore, HM was compared with Nimodipine in two studies, Huperzine A in one study, cerebroprotein hydrolysate in one study, and placebo in three studies. All of these studies concluded that HM is better than the control intervention.

### 4.8. Herbal Medicine as an Adjunct Therapy (Table 3)

Four trials evaluated HM as an adjunct therapy for VaD. Two of them evaluated the adjunct effect of the CHM decoction BuYangHuanWuTang. Wang compared it with the coadministration of Piracetam and Nimodipine; Yan compared it with the co-administration of Aniracetam, Nimodipine, together with the injection of cerebroprotein hydrolysate. Shi and Wang studied the CHM decoction which, according to TCM theory, could “tonify the kidney, activating blood,” and tested its adjunct effect with nimodipine + hydergine (In TCM theory, the brain is considered an outgrowth of “kidney” energy. Neurodegenerative disorders such as dementia are caused by stagnation of “blood,” accumulation of “phlegm,” and deficiency of the “kidney.” In order to resist or halt the condition, TCM treatment targets the nourishment of the kidney by means of “kidney invigorating,” “blood activating” and “phlegm dissipating” herbal decoctions [110]. Guo et al. [53] studied another CHM BuNaoTongQiao decoction, which possesses nootropic properties, according to TCM theory, and compared it with Duxil. All these studies reported that when HM is used together with Western medications, both the efficacy and safety of OM could be enhanced.

### 4.9. Adverse Events and Withdrawal (Table 3)

Among those 43 studies which tested HM as monotherapy, 25 studies did not report any cases of withdrawal. Ten studies claimed they did not observe any adverse events in groups treated with HM. Eight studies reported a number of mild adverse events, such as mouth dryness, sore throat, constipation, nausea, loss of appetite, and dyspepsia. These adverse events could be resolved without treatment. Serious adverse events were not observed. Occurrence of adverse events remained unclear in the four studies which tested HM as adjunct therapy.

The dropouts or withdrawals were unclear in 44 out of 47 studies. Wu et al. [83] reported two dropouts in the course of intervention. Liu et al. [71] reported 46 dropouts during his trial. Cui et al. [77] reported five dropouts.

## 5. Discussion

### 5.1. A Wide Variety of Herbal Remedies

Thirty-one out of 47 studies tested herbal mixtures prepared in the form of granules or capsules. Fifteen studies tested their herbal mixtures in the form of decoctions. One study tested the extract from a single herb. As some of the studies tested the same herbal mixture, altogether 42 different herbal mixtures were tested among these 47 studies (Table 4). These herbal mixtures or extracts, according to the TCM theory, have the ability to “tonify the “kidney,” activate blood.” Despite the absence of pharmacological studies to verify their safety, these studies reported encouraging effects and high safety profiles. Upon further analysis of the constitutional ingredients in these herbal formulas, we ranked the 30 most commonly used herbal constituents together according to the dosages (Table 5).

The first five in descending order of frequency of use are Rhizoma Chuanxiong (Chuanxiong in Chinese), Radix Polygoni Multiflori (Heshouwu in Chinese), Radix Astragali (Huangqi in Chinese), Radix Ginseng (Renshen in Chinese), and Rhizoma Acori Tatarinowii (Shichangpu in Chinese). 

Rhizoma Chuanxiong, originates from the plant *Ligusticum chuanxiong* Hort., which is used in TCM to “remove blood stasis.” Chemical analysis shows that it possesses an alkaloid named ligustrazine, which has antioxidant, anti-inflammatory, antifibrosis, and immune-modulative properties [111]. A clinical study is being carried out to evaluate its effect on patients' recovery from cerebral vascular accidents [112]. The root of *Polygonum multiflorum* Thunb. (Radix Polygoni Multiflori in English, the Chinese name is Heshouwu), commonly known as fleece flower root, is another popular HM used to treat premature aging and dementia. Past studies have shown it to have activity that may contribute to cardiovascular protection [113]. Long-term pretreatment with it may protect the brain against focal cerebral ischemia [114]. One animal study also suggests that it has anti-oxidant properties [115], with the capacity to prevent cognitive deficits [116], possibly even to promote learning and enhance memory [117]. A medical team in Taiwan is proposing a phase II clinical trial to assess the efficacy and safety of a new drug derived from it [118]. Radix Astragali (Huangqi), from *Astragalus membranaceus* (Fisch.) Bge. var. *mongholicus* (Bge.) Hsiao, a commonly used herb to “vitalize spleen Qi” and “treat circulatory disorders” in TCM, possesses various components (astragalus saponins, astragalus polysaccharide) demonstrated to have anti-oxidation properties. These, together with its anti-cholinergic property, have been suggested to be the source of its significant anti-dementia effect [119]. A clinical trial is being carried out to determine the effect of an HM capsule with Radix Astragali (Huangqi in Chinese) as the main constituent on ischemic stroke [112]. Radix Salviae Miltiorrhizae (Danshen in Chinese), the root of *Salvia miltiorrhiza* Bge., is used to “activate blood and resolve stasis” according to TCM. A laboratory study of its triterpenoids-enriched extract revealed that its antiatherogenic property was mediated by an anti-inflammatory mechanism [120]. Another animal study of Radix Salviae Miltiorrhizae (Danshen) reports it to reduce the area of cerebral infarct in ischemia-reperfusion injured rats, suggesting it has potential in the treatment of cerebral infarct in humans [121]. Radix Ginseng (Rensheng), the root of *Panax ginseng* C.A. Mey., is a popular notifying herb in TCM, and its ginsenosides have been found to have protective effects on memory via antiapoptosis in a rat model with vascular dementia [122], and to stimulate angiogenesis and tissue regeneration [123], suggesting that it has potential to help VaD patients. In a Korean clinical study, Radix Ginseng was reported to be clinically effective in improving the cognitive performance of AD patients [124]. Rhizoma Acori Tatarinowii (Shichangpu), also named grassleaf or sweet-flag rhizome, the rhizome of *Acorus tatarinowii* Schott., is used in TCM for resuscitation after coma. Pharmacological studies suggest this effect may be due to the increase in permeability of the blood-brain barrier [125]. In another pharmacological study, the fruit of *Cornus officinalis* Sieb. et Zucc. (Shanzhuyu in Chinese), which is used in TCM to “tonify the kidney,” was found to possess an extract that has protective effects against oxidative stress-induced neurotoxic processes [126]. Other experimental reports have indicated that the triterpenoid saponins from the roots of *Polygala tenuifolia* Willd. (Yuanzhi) possess neuroprotective effects [127, 128]. Study on extracts of *Alpiniae Oxyphyllae* Miq. (Fructus Alpinae Oxyphyllae in English, the Chinese name is Yizhi) have found evidence that it protects neurons against ischemia-induced cell death [129] and that it prevents glutamate-induced apoptosis in cortical neurons [130]. In another study, it was reported that *Rhizoma Polygonati* (known as Huangjing in Chinese, used in TCM as a notifying agent) could improve learning and memory in a scopolamine-induced mouse model of dementia by reducing the damaging effects of cerebral ischemia and anti-oxidation, having similar effects to those provided by vitamin E [131]. In a study to examine the anti-oxidative and neuroprotective effects of *Paeonia lactiflora* Pall. (Baishao in Chinese), it was found to suppress the hydrogen peroxide-induced apoptosis in PC12 cells, suggesting that it could be a new antioxidant useful in the prevention of neuronal diseases [132]. Rhizoma Gastrodiae from *Gastrodia elata* Bl. (Tianma in Chinese), a classic HM used to “extinguish wind and arrest convulsions” in TCM theory, possesses vasodilating [133], anti-inflammatory, and antiangiogenic activities [134], suggesting a potential VaD treatment. The total alkaloids found in Radix Codonopsis (Dangshen in Chinese used in TCM to “tonify Qi”) have been reported to potentiate neurite outgrowth induced by nerve growth factor in PC12 cells [135]. Glossy privet fruit, from *Ligustrum lucidum* Ait. (Nüzhenzi in Chinese), a kidney-tonifying HM, can inhibit cell apoptosis by reducing apoptotic signals induced by cerebral ischemia/hypoxia [33].

### 5.2. Study Weaknesses

Though all the studies reported promising results of HM in the treatment of VaD, they demonstrated a number of weaknesses as well. The evidence drawn from the studies was insufficient for us to confirm the safety and efficacy of HM, because of the following issues

1. The sample sizes of the studies ranged from 18 to 300, and none of them reported sample size calculations, as suggested by the CONSORT statement. Treatment effects can be exaggerated when sample size is inappropriate, and thus the results of these studies may not be conclusive.
2. Different diagnostic criteria were used in the studies. Some of these criteria were even self-derived and their validities remained unknown. This produced much discrepancy.
3. Differences in the baseline characteristics of the subjects limit the extent to which results can be compared with each other.
4. Though all of the studies claimed to have participants allocated randomly, only a few reported the method of randomization. For those studies without detailed descriptions of randomization, we could not rule out the possibility of bias. Furthermore, unclear descriptions of allocation concealment, dropouts, and intention-to-treat analysis further hamper the ability to assess the validity of the evidence reported by these studies.
5. Outcome measures varied and were incomplete in the studies. Some investigators employed self-developed scales, which could not be, or had not been, independently evaluated for their sensitivity and specificity. The validity is further questionable due to insufficient or inappropriate statistical treatment. Though meta-analysis techniques such as vote-counting may have been used for the analysis of the data, we avoid to do so because (1) the statistical significance or size of the results of the individual studies are ignored; and (2) vote-counting takes no account of the differential weights given to each study. [136]
6. Different HM were tested in the 47 studies included here, with great variation in terms of composition, dosage, and duration of interventions. This renders comparison of the studies impossible, and thus quantitative analysis could not be carried out.
7. A number of studies (30 out of 47) did not mention safety issues. The investigators of these studies may have underestimated possible adverse events, and the safety of HM in these studies could not be guaranteed.

### 5.3. Implications for Further Studies

Regarding the published studies, methodology quality is the leading should concern. It is recommended that future clinical studies follow the guidelines as suggested by CONSORT to minimize bias as well as to ensure high validity, statistically reliable results and to permit comparison with other studies. Researchers should explicitly report methods for calculation of sample size. Widely recognized diagnostic criteria and outcome measures should be used. It is highly recommended to incorporate medical imaging techniques (such as perfusion computed tomography) to confirm the diagnosis of VaD. Appropriate statistical analyses should be carried out for baseline data and outcome results; long-term followup is also recommended and highly desirable.

Our review has identified the individual herbs that appear most frequently in formulas for VaD. The top five are Rhizoma Chuanxiong (Chuanxiong), Radix Polygoni Multiflori (Heshouwu), Radix Astragali (Huangqi), Radix Ginseng (Renshen), and Rhizoma Acori Talarinowii (Shichangpu). The clinical efficacy and safety of these herbs, over centuries of use and during recent controlled studies, are a powerful combination of attributes. We believe that further high-quality clinical studies on these individual constituents, as well as the herbal mixtures resulted, could lead to the discovery of new drugs for effective treatment and prevention of VaD.

## 6. Conclusion

Currently available RCTs suggested that HM might be more effective and safer than OM for treatment of VaD. However, these studies have a number of weaknesses, mainly due to their methodological insufficiencies. With regard to the reports that did meet our selection criteria, the results indicated that HM, in a predominance of instances, can be superior to OM and useful in the treatment of VaD. Further multicenter trials with large sample sizes, high methodological quality, and standardized HM ingredients are needed to confirm the value of HM in treating VaD, in order to establish specific clinical recommendations.

## Figures and Tables

**Table 1:** Search strategy.

1	exp Plant Extracts/or exp Drugs, Chinese Herbal/or exp Plants, Medicinal/or exp Medicine, Chinese Traditional/or exp China/or chinese medicine.mp. or exp Medicine, Oriental Traditional/or exp Phytotherapy/
2	drugs non prescription.mp. or exp Drugs, Non-Prescription/
3	medicinal herbs.mp
4	herbs medicinal.mp.
5	drugs non prescription.mp. or exp Drugs, Non-Prescription/
6	alternative medicine.mp. or exp Complementary Therapies/
7	complementary medicine.mp.
8	Phytotherapy/or Plants, Medicinal/or Plant Extracts/or Herb-Drug Interactions/or herbs.mp. or Drugs, Chinese Herbal/or Plant Preparations/
9	exp Phytotherapy/or exp Plants, Medicinal/or exp Plant Extracts/or exp Herb-Drug Interactions/or exp Alkaloids/or herbs.mp. or exp Drugs, Chinese Herbal/or exp Plant Preparations/
10	1 or 2 or 3 or 4 or 5 or 6 or 7 or 8 or 9
11	randomized controlled trials.mp. or exp Randomized Controlled Trials/
12	exp Random Allocation/or exp Clinical Trials/or exp Double-Blind Method/or double blind.mp. or exp Placebos/
13	single blind.mp. or exp Single-Blind Method/
14	clinical trials.mp.
15	prospective studies.mp. or exp Prospective Studies/
16	follow up studies.mp. or exp Follow-Up Studies/
17	11 or 12 or 13 or 14 or 15 or 16
18	exp Mental Retardation/or exp Dementia/or exp vascular dementia/or progressive brain disorder.mp. or exp Memory Disorders/
19	(vascular dementia).mp.
20	18 or 19
21	10 and 17 and 20

**Table 2:** Methodological quality of studies (CONSORT checklist).

			Reported page number of each item^∗1^																					
			Abstracts		Method					Randomization					Results							Discussion		
Number	Author	Year	Title and abstract	Introduction	Participant	Intervention	Objective	Outcome	Sample size	Sequence generation	Allocation concealment	Implementation	Blinding	Statistical methods	Participant flow	Recruitment	Baseline data	Numbers analyzed	Outcomes and estimation	Ancillary analyses	Adverse events	Interpretation	Generalisability	Overall evidence
			1	2	3	4	5	6	7	8	9	10	11	12	13	14	15	16	17	18	19	20	21	22
1	Wan et al.	1998	25	25	25	25	24	25	U	U	U	U	U	U	U	U	25	26	26	U	U	27	U	27
2	Zhao et al.	1999	585	585	585	586	585	586	U	U	U	U	U	586	U	585	585	585	586	U	587	587	587	587
3	Ji	2000	10	10	10	11	U	11	U	U	U	U	U	U	U	10	10	U	11	U	U	11	U	11
4	Lu et al.	2000	290	290	290	290	290	290	U	U	U	U	U	290	U	U	290	290	290	U	U	290	U	290
5	Luo	2001	470	U	470	470	470	471	U	U	U	U	470	471	U	470	470	472	472	472	U	472	473	473
6	Zhang et al.	2001	U	51	51	51	U	51	U	U	U	U	U	U	U	51	51	52	52	U	52	52	52	52
7	Zhou and Yi	2001	14	14	14	14	14	14	U	U	U	U	U	U	U	U	14	15	15	U	U	15	U	15
8	Cao et al.	2002	80	80	80	80	80	80	U	U	U	U	80	81	U	80	80	81	81	U	81	81	U	81
9	Hong et al.	2002	U	3	3	3	U	3	U	U	U	U	U	3	U	3	3	4	4	4	U	5	5	5
10	Huang et al.	2002	301	301	301	302	301	302	U	U	U	U	U	302	U	U	302	301	302	U	U	303	303	303
11	Liu et al.	2002	526	526	526	526	526	526	U	U	U	U	U	U	U	526	526	U	527	U	U	527	U	527
12	Wang et al.	2002	U	295	296	296	U	296	U	U	U	U	U	U	U	295	295	296	296	U	U	296	U	296
13	Yang et al.	2002	48	48	49	49	48	49	U	U	U	U	48	49	U	U	48	49	49	U	U	50	51	51
14	Cai et al.	2003	482	482	482	482	482	482	U	482	U	U	U	483	U	U	482	483	483	U	483	483	483	483
15	Guo et al.	2003	U	931	931	931	U	931	U	U	U	U	U	U	U	U	931	931	931	U	U	931	U	931
16	Jia et al.	2003	20	20	20	20	20	21	U	U	U	U	U	21	U	20	20	21	21	U	21	22	U	22
17	Cheng et al.	2004	16	16	16	16	16	16	U	U	U	U	U	16	U	U	16	17	17	U	U	17	17	17
18	Liao et al.	2004	112	112	112	113	112	113	U	U	U	U	U	113	U	113	112	113	113	U	113	113	114	114
19	Shen and Du	2004	41	41	42	42	41	42	U	U	U	U	U	42	U	42	42	42	43	U	U	43	43	43
20	Wang et al.	2004	679	679	679	680	679	680	U	680	680	680	680	680	681	U	679	680	680	U	681	681	681	681
21	Wang et al.	2004	1691	1691	1691	1692	1691	1692	U	U	U	U	U	1692	U	1691	1691	1692	1692	U	U	1693	U	1693
22	Wu et al.	2004	3	3	3	3	3	3	U	U	U	U	3	3	U	U	3	4	4	U	4	4	U	4
23	Yu et al.	2004	424	424	424	424	424	424	U	U	U	U	424	U	U	424	424	425	425	U	425	425	425	425
24	Zhao	2004	8	9	9	9	8	9	U	U	U	U	U	9	U	9	9	9	9	U	9	9	U	10
25	Feng et al.	2005	520	520	520	520	520	520	U	U	U	U	U	521	U	520	520	521	521	U	U	521	522	521
26	Liu	2005	50	50	50	50	50	50	U	U	U	U	U	50	51	50	51	51	51	U	51	51	51	51
27	Liu et al.	2005	1052	1052	1052	1052	U	1052	U	U	U	U	U	1053	U	1053	1052	1053	1053	U	1054	1054	1054	1054
28	Liu and Chen	2005	18	18	18	19	18	19	U	U	U	U	U	19	U	U	18	19	19	U	U	20	U	20
29	Tang et al.	2005	426	426	426	427	426	427	U	U	U	U	U	427	U	426	426	427	427	U	427	427	427	427
30	Wang et al.	2005	93	U	93	94	93	94	U	94	94	U	94	94	U	93	94	94	94	U	95	94	U	94
31	Wang, Chen and Bai	2005	3	3	3	3	3	4	U	U	U	U	U	4	U	3	3	3	4	U	U	4	5	5
32	Wang et al.	2005	260	260	261	261	260	261	U	U	U	U	261	261	U	U	260	261	261	U	261	262	U	262
33	Wang	2005	40	40	40	40	40	40	U	U	U	U	U	40	U	40	40	40	41	U	U	41	41	41
34	Zhou et al.	2005	11	11	11	11	11	12	U	U	U	U	U	12	U	11	11	12	12	U	U	13	U	13
35	Gao	2006	14	14	14	14	14	15	U	U	U	U	U	U	U	14	14	15	15	U	U	16	U	16
36	Hao et al.	2006	424	424	424	424	424	424	U	U	U	U	U	425	U	U	424	425	425	U	425	425	U	425
37	Li et al.	2006	48	48	48	48	U	48	U	U	U	U	U	48	U	48	48	48	48	U	U	49	49	49
38	Mou	2006	1607	1607	1607	1607	1607	1607	U	U	U	U	U	U	U	U	1607	1607	1607	U	1607	U	U	1607
39	Shi and Wang	2006	200	200	200	200	200	200	U	200	U	U	200	200	U	U	200	201	201	U	U	201	201	201
40	Zhang and Lu	2006	680	680	680	680	680	681	U	U	U	U	U	U	U	U	680	681	681	U	U	681	681	681
41	Chen et al.	2007	866	866	866	867	866	867	U	867	U	U	867	867	U	866	866	867	867	U	U	868	868	868
42	Cui et al.	2007	64	64	64	64	64	64	U	64	U	U	U	65	U	64	64	U	65	U	U	65	U	65
43	He	2007	60	U	60	60	U	60	U	U	U	U	60	60	U	U	60	60	60	U	61	61	U	61
44	Jin et al.	2007	1657	1657	1657	1658	1657	1658	U	U	U	1658	1658	1658	U	U	1657	1658	1658	U	1659	1659	1659	1659
45	Yan	2007	41	U	41	41	41	41	U	U	U	U	U	U	U	U	41	41	41	U	U	41	41	42
46	Chang	2008	241	240	240	241	240	241	U	U	U	U	U	U	U	240	241	241	241	U	241	241	U	241
47	Li et al.	2008	369	369	369	370	369	370	U	U	U	U	U	370	U	369	369	370	370	U	371	371	371	371

Key ^∗1^: U = the relevant item was not found in the paper.

**Table 3:** Study properties.

	Study	Design	Sample and characteristics	Diagnostic criteria	Herbal intervention	Control	Outcome measures	ITT^∗1^	Drop out^∗2^	ADR^∗3^
1	Wan et al. [63], China Monotherapy Unclear center	Randomized: method not mentioned; double blinding not mentioned; parallel design; 2 months duration	68 VaD patients, age: 58–82; duration: 4.37–15.85 yr	DSM-3-R, HDS, HIS, “TCM dementia differential criteria”^∗a^	Fucong 150 mL, bid (37)	Piracetam 0.8 g, tid (31)	MMSE, HDS, BEAM, hemodynamic changes	N	N	U
2	Zhao et al. [74], China Monotherapy Single center	Randomized: method not mentioned; single blind; parallel design; 2 months duration	46 VaD patients; age: 57–76; duration: 6 m –1 yr	DSM-4, HIS, HDS, MMSE, “Protocol for new herbal drugs study on dementia”^∗ b^	Xianlong 2.7 g, bid (24)	Hydergine 3 mg, bid (22)	TCD, hemodynamic changes	N	N	U
3	Ji [59], China Monotherapy Unclear center	Randomized: method not mentioned; double blinding not mentioned; parallel design; 2 months duration	68 VaD patients; age: 58–82; duration: 1.45-3.24 yr	DSM-3-R, HDS, HIS, “TCM dementia differential criteria”^∗ a^	Dangguishaoy ao bid (37)	Piracetam 0.8 g, tid (31)	MMSE, HDS	N	N	U
4	Lu et al. [90], China Monotherapy Unclear center	Randomized: method not mentioned; double blinding not mentioned; parallel design; 60 days duration	50 VaD patients; age: 56–82; duration: 2–6 yr	DSM-3-R, MMSE, “TCM diagnostic criteria, differentiation and outcome measures on senile dementia”^∗ c^	Shentong 10 g, tid (30)	Hydergine 1 mg, tid (20)	MMSE, BBS	N	N	U
5	Luo et al. [55], China Monotherapy Multicenter	Randomized: method not mentioned; double blind: method not mentioned; parallel design; 75 days duration	68 VaD patients; age: 49–79; duration: 0.99–2.07 yr	DSM-4, ICD 10, HIS, “TCM diagnostic criteria, differentiation and outcome measures on senile dementia”^∗ c^	Shenmayizhi 1 g, tid (35)	Hydergine 1 mg, tid (33)	MMSE, ADL, BEAM, Neurological deficits	N	N	U
6	Zhang et al. [51], China Monotherapy Unclear center	Randomized: method not mentioned; double blinding not mentioned; parallel design; 3 months duration	61 VaD patients; age: 60–77; duration: 6 m–2.5 yr;	NINDS-AIREN, HIS, HDS, “protocol for new herbal drugs study on dementia”^∗ b^	Jiannaotongluo 1.6 g, tid (30)	Aniracetam 0.3 g, tid (31)	HDS, hemodynamic changes	N	N	U
7	Zhou and Yi [64], China Monotherapy Single center	Randomized: method not mentioned; double blinding not mentioned; parallel design; 3 months duration	46 VaD patients; age: 60–80; duration: 8 m–3 yr	DSM-3, “protocol for new herbal drugs study on dementia,”^∗b^ “protocol for new herbal drugs study on stroke”^∗ b^	Yinaoling 40 mL, bid (23)	Piracetam 0.8 g tid (23)	HDS, FAQ, CCSE	N	N	U
8	Cao et al. [82], China Monotherapy Unclear center	Randomized: method not mentioned; double blind: method not mentioned; parallel design; 60 days duration	53 VaD patients; age: 58–75; duration: 3 m–12 m	DSM-4	Congsheng tid (25)	Hydergine tid (28)	MMSE, BBS, TCD, EKG, SELF	N	N	N
9	Hong et al. [54], China Monotherapy Single center	Randomized: method not mentioned; double blinding not mentioned; parallel design; 60 days duration	86 VaD patients; age: 45–76; duration: 5 m–3 yr	DSM-4, MMSE, “TCM diagnostic criteria, differentiation and outcome measures on senile dementia,”^∗ c^ CORELATION, HIS	Shouxing tid (48)	Piracetam tid (38)	MMSE, ADL	N	N	U
10	Huang et al. [91], China Monotherapy Unclear center	Randomized: method not mentioned; double blinding not mentioned; parallel design; 3 months duration	58 VaD patients; age: 57–79; duration: 6 m–3 yr	DSM-4, MMSE, ADL, HIS, “TCM diagnostic criteria, differentiation and outcome measures on senile dementia”^∗ c^	Naohuandan 62 g/d (28)	Piracetam tid (28)	MMSE, ADL, E2, T, hemodynamic changes	N	N	U
11	Liu et al. [65], China Monotherapy Unclear center	Randomized: method not mentioned; double blinding not mentioned; parallel design; 2 months duration	64 VaD patients; age: 54–81; duration: 0.8–3 yr	DSM-4, HIS, CT, MRI	Tongqiaohuoxue- buyanghuanwu bid (36)	Duxil 1 tablet bid (28)	MMSE, HDS, hemodynamic changes	N	N	U
12	Wang et al. [52] Monotherapy Single center	Randomized: method not mentioned; single blind: method not mentioned; parallel design; 30 days duration	300 VaD patients; age: 52–83; duration: 7 m–7 yr	DSM-3-R, DSM-4, MMSE, “TCM diagnostic criteria, differentiation and outcome measures on senile dementia,”^∗ c^ HIS, CT, MRI	Yizhitongluo 0.9–1.2 g TID (200)	Piracetam 0.8 g tid (100)	MMSE, HDS, GDS, ADL, hemodynamic changes, SELF	N	N	U
13	Yang et al. [80], China Monotherapy Single center	Randomized: method not mentioned; single blind: method not mentioned; parallel design; 2 months duration	90 VaD patients; age: 50–81; duration: 3 M–6.5 yr	DSM-4-R, ICD10, CDR, MMSE, HDS-R, CORNELL, “protocol for new herbal drugs study on dementia”^∗ b^	Zhinao 1.5 g tid (60)	Hydergine 2 mg tid (30)	MMSE, HDS, ADL, neurological deficits, hemodynamic changes, TCD, EEG, “TCM diagnostic criteria, differentiation and outcome measures on senile dementia”^∗c^	N	N	U
14	Cai et al. [72], China Monotherapy Center unclear	Randomized: computer generated sequence; single blind: method not mentioned; parallel design; 3 months duration	63 VaD patients; age: 65–78; duration: 3 m–94 m	DSM-4-R, MMSE, HIS, CT, MRI	Kangxing 0.9 g tid (33)	Hydergine 2 mg tid (30)	MMSE, ADL, hemodynamic changes, “protocol for new herbal drugs study on stroke” *中藥* *新* *藥治療* *癡呆的* *臨* *床研究指導原則*	N	Y	N
15	Guo et al. [53], China Adjunct therapy Center unclear	Randomized: method not mentioned; double blinding not mentioned; parallel design; 2 months duration	53 VaD patients; age: 55–73; duration: 1 yr–5 yr	DSM-4, “protocol for new herbal drugs study on dementia,”^∗ b^ “protocol for new herbal drugs study on stroke”^∗ b^	Bunaotongqiao + Duxil 1 tablet bid (28)	Duxil 1 tablet bid (25)	MMSE, HDS, hemodynamic changes,	N	N	U
16	Jia et al. [92], China Monotherapy Single center	Randomized: method not mentioned; single blind: method not mentioned; parallel design; 60 days duration	162 VaD patients; age: 54–71; duration: 0.8–3 yr	DSM-4, CDR, imaging, HDS	Luoshukang 1.5–2.5 g tid (108)	Duxil 1 tablet bid (54)	MMSE, BBS, “protocol for new herbal drugs study on dementia”^∗ b^	N	N	N
17	Cheng et al. [93] Monotherapy Single center	Randomized: method not mentioned; single blind: method not mentioned; parallel design; 2 months duration	36 VaD patients; age: 62–83; duration: 0.5–9 yr	DSM-4-R, HIS, CT, MMSE, FAQ, HDS-R, ADL	Naozhitong 4 pcs tid (18)	Nimodipine 20 mg tid (18)	MMSE, FAQ, HDS-R, ADL, NO	N	N	U
18	Liao et al. [73], China Monotherapy Single center	Randomized: computer-generated sequence; single blind: method not mentioned; parallel design; 2 months duration	60 VaD patients; age: 71–74; duration: 3.2–5.6 hr	DSM-4, CT, MRI, HIS, MMSE, HDS, “TCM diagnostic criteria, differentiation and outcome measures on senile dementia”^∗c^	Shoulingjiannao 0.9 g tid (32)	Hydergine 1 mg tid (28)	MMSE, HDS, “protocol for the selection of anti-aging herbal medicine and the corresponding outcome measures”^∗ d^	N	N	N
19	Shen and Du [94], China Monotherapy Single center	Randomized: method not mentioned; single blind: method not mentioned; parallel design; 3 months duration	70 VaD patients; age: 65–77; duration: 6 m –3 yr	DSM-4, NINDS-AIREN, CT, MRI, “protocol for new herbal drugs study on dementia”^∗ b^	Bushenjianpiy-angxuehuoxue 0.9 g tid (40)	Hydergine 2 mg tid (30)	MMSE, ADL, ET, NO, HCY, E2, T	N	N	U
20	Wang et al. [95], China Monotherapy Single center	Randomized: method not mentioned; double blind: details given; crossover design; 7 months duration (3 m + 1 m wash out + 3 m)	18 VaD patients; age: 54–83; duration: 1–7 yr	DSM-4, CT, MRI, HDS, MMSE, ADL-R, “protocol for new herbal drugs study on dementia”^∗ b^	Shenlong 180 mL bid (18)	Placebo 180 mL bid (18)	HDS, MMSE-R, ADL-R, “protocol for new herbal drugs study on dementia” ^∗b^	N	N	Y (2, mouth dryness, sore throat)
21	Wang et al. [96], China Monotherapy Single center	Randomized: method not mentioned; single blind: method not mentioned; parallel design; 3 months duration	100 VaD patients; age: 50–78; duration: 0.7–1.8 hr	DSM-4, HIS, ADL, MMSE, SDS, “TCM diagnostic criteria, differentiation and outcome measures on senile dementia”^∗ c^, “protocol for new herbal drugs study on dementia”^∗ b^	Huitian 0.8 g tid (50)	Piracetam 08. g tid (50)	MMSE, ADL, hemodynamic changes, “protocol for new herbal drugs study on dementia” ^∗b^	N	N	U
22	Wu et al. [83], China Monotherapy Center unclear	Randomized: method not mentioned; double blind: method not mentioned; parallel design; 30 days duration	46 VaD patients; age: 62–77; duration: 0.8–4.7 yr	DSM-4, CCDVD, CDSVD-R, MMSE, HIS	Extract from *Herba Cistanches* 2 tablets tid (23)	Hydergine 2 tablet tid (23)	MMSE, BBS, ADL, hemodynamic changes	N	2	N
23	Yu et al. [66], China Monotherapy Single center	Randomized: method not mentioned; single blind: method not mentioned; parallel design; 14 days duration	123 VaD patients; age: 57–74; duration: 0.4–1.4 yr	DSM-4, “TCM diagnostic criteria, differentiation and outcome measures on senile dementia,”^∗ c^ CT	Fucong 30 mL od (72)	Piracetam 0.8 g tid + vitamin E 0.1 g tid (51)	SOD, LPO, TG, TCH, HDL, EEG	N	N	Y (2, mouth dryness, sore throat)
24	Zhao [97], China Monotherapy Center unclear	Randomized: method not mentioned; double blinding not mentioned; Parallel design; 2 months duration	90 VaD patients; age: 49–81; duration: 0.5–3.5 yr	DSM, CT, MRI, clinical presentation	Jiannaoqingxin od (50)	Duxil 1 tablet bid (40)	MMSE, MMSE-R, hemodynamic changes, HDS	N	N	N
25	Feng et al. [86], China Monotherapy Single center	Randomized: method not mentioned; double blinding not mentioned; parallel design; 6 months duration	50 VaD patients; age: 59–82; duration: 2–5 yr	Portera-sanchey	Yizhi bid (30)	Piracetam 1.6 g tid (20)	MMSE, BBS, ADL	N	N	U
26	Liu [71], China Monotherapy Single center	Randomized: method not mentioned; no blinding is applied; parallel design; 2 months duration	142 VaD patients; age: 64–68; duration: 1.9–4.2 yr	DSM-R, CT, MRI, HIS	Bushenyinao 3 g tid (98)	Cerebroprot-ein Hydrolysate iv. 30 mL/day (44)	HDS, hemodynamic changes	N	46	Y
27	Liu et al. [60], China Monotherapy Single center	Randomized: method not mentioned; double blinding not mentioned; parallel design; 2 months duration	92 VaD patients; age: 45–80; duration: 1–12 yr	DSM, MMSE, ADL, CDR, HIS, “protocol for new herbal drugs study on dementia”^∗ b^	Huatuozaizao 8 g tid (52)	Duxil 1 tablet bid (40)	MMSE, ADL, TC, TG, HDL-C, ET, NO, SELF, “protocol for new herbal drugs study on dementia” ^∗b^	N	N	N
28	Liu and Chen [74], China Monotherapy Single center	Randomized: computer generated sequence; double blinding not mentioned; parallel design, 8 weeks duration	86 hospitalized VaD patients; age: 60–79; duration: 4 m–6.5 yr	DSM-4, HDS, HIS	Buyanghuanwu 12 g bid (43)	Hydergine 1 mg bid (43)	HDS, FAQ, hemodynamic changes, MQ, “protocol for new herbal drugs study on dementia”∗b	N	N	U
29	Tang et al. [75], China Monotherapy Single center	Randomized: computer generated sequence; double blinding not mentioned; parallel design; 3 months duration	80 VaD patients; age: 53–80; duration: 6 m–12 yr	DSM-3-R, MMSE, CT, MRI, HIS, “TCM manual for neurological diseases”^∗ e^	Bushenhuoxie bid (40)	Duxil 1 tablet bid, Nimodipine 30 mg tid (40)	MMSE, ADL, WBHSV, WBLSV, PV, HCT, “TCM diagnostic criteria, differentiation and outcome measures on senile dementia”^∗ c^	N	N	N
30	Wang et al. [56], China Monotherapy Unclear center	Randomized: method not mentioned; double blind: details given; crossover design; 7 months duration (3 m + 1 m wash out + 3 m)	36 VaD patients; age: 52–83; duration: 1–7 yr.	DSM-4, ADL-R	Shenlong 180 mL bid (36)	Placebo bid (36)	MMSE-R, BBS, HDS, ADL-R	N	N	Y (4, sore throat, mouth dryness)
31	Wang et al. [98], China Monotherapy Single center	Randomized: method not mentioned; double blinding not mentioned; parallel design; 3 months duration	140 VaD patients; age: 50–78; duration: 0.9–1.8 yr	DSM-4, HIS, ADL, MMSE, SDS, “protocol for new herbal drugs study on dementia,”^∗ b^ “TCM diagnostic criteria, differentiation and outcome measures on senile dementia” ^∗c^	Jiannaocongming 100 mL tid (100)	Piracetam 0.8 g tid (40)	MMSE, ADL, hemodynamic changes	N	N	U
32	Wang et al. [99], China Monotherapy Single center	Randomized: method not mentioned; double blinding not mentioned; parallel design; 6 months duration	80 hospitalized VaD patients; age: 46–78; duration: 6–122 m	DSM-4, NINDS-AIREN, MMSE, HDS, FAQ, HIS, “protocol for new herbal drugs study on dementia”^∗b^	Tongxinluo 3 pcs tid (40)	Huperzine A 0.1 mg bid (40)	MSME, HDS, FAQ, “TCM diagnostic criteria, differentiation and outcome measures on senile dementia”^∗ c^	N	N	Y (nausea, decrease in appetite, etc.)
33	Wang [100], China, Adjunct therapy Single center	Randomized: method not mentioned; double blinding not mentioned, parallel design; 45 days duration	66 VaD patients; age: 51–76; duration: 0.5–6 yr	DSM-4-R, MMSE, HDS, CT, MRI	Buyanghuanwu od + Piracetam 1.2 g bid, Nimodipine 20 mg tid (33)	Piracetam 1.2 g bid, Nimodipine 20 mg tid (33)	HDS-R	N	N	U
34	Zhou et al. [101], China Monotherapy Single center	Randomized: method not mentioned; double blinding not mentioned; parallel design; 3 months duration	62 VaD patients; age: 59–76; duration: 0.5–6 yr	CCMD-2-R, DSM-4, MMSE, HDS, HIS	Yiqifuzhi 46 g tid (30)	Piracetam 0.8 g tid (32)	Hemodynamic changes, SOD, MDA, “protocol for new herbal drugs study on dementia”^∗ b^	N	N	U
35	Gao [67], China Monotherapy Single center	Randomized: method not mentioned; double blinding not mentioned; parallel design; 3 months duration	98 VaD patients; age: 54–75; duration: 0.2–1.8 yr	DSM-4, “TCM diagnostic criteria, differentiation and outcome measures on senile dementia”^∗ c^, ADL, MMSE	Shumaiyinao 1.2 g tid (58)	Piracetam 1.2 g tid (40)	MMSE, hemodynamic changes, Vmin, Qmin, RI	N	N	U
36	Hao et al. [102], China Monotherapy Single center	Randomized: method not mentioned; double blinding not mentioned; parallel design; 6 months duration	100 VaD patients; age: 48–81; duration: 7–118 m	ICD10, CT, MMSE, IADL, HIS	Tongxinluo 3 pcs tid (50)	Piracetam 0.8 g tid (50)	MMSE, NPI, IADL, HIS	N	N	Y (16, GI discom-fort)
37	Li et al. [61], China Monotherapy Single center	Randomized: method not mentioned; double blinding not mentioned; parallel design; 3 months duration	60 VaD patients; age: 45–80; duration: 1–12 yr	DSM-4, ADL, MMSE, HIS, “protocol for new herbal drugs study on dementia”^∗ b^	Tongmaiyizhi 8 g tid (30)	Duxil 1 tablet bid (30)	MMSE, ADL, “protocol for new herbal drugs study on dementia”^∗ b^	N	N	U
38	Mou [68], China Monotherapy Unclear center	Randomized: method not mentioned; double blinding not mentioned; parallel design; 60 days duration	60 VaD patients; age: 57–89; duration: 2.5–4 yr	DSM-4, HDS	Self-derived CHM 16 g 100 mL bid (30)	Nimodipine 30 mg tid (30)	MMSE	N	N	U
39	Shi and Wang [76], China Adjunct therapy Single center	Randomized: computer generated sequence; double blind: method not mentioned; parallel design; 3 months duration	78 VaD patients; age: 50–80+; duration: 5 m–1.5 yr	DSM-4, “TCM diagnostic criteria, differentiation and outcome measures on senile dementia,”^∗ c^ “Diagnostic manual for geriatrics”^∗ f^	Self-derived CHM 2 bid + nimodipine 40 mg tid, hydergine 2 mg tid (46)	nimodipine 40 mg tid, hydergine 2 mg tid (32)	ADL, MMSE	N	N	U
40	Zhang and Lu [69], China Monotherapy Unclear center	Randomized: method not mentioned; double blinding not mentoned; parallel design; 3 months duration	72 VaD patients; age: 57–71; duration: 0.3–5.6 yr	DSM-4, ICD10, HIS, HDL, TC	Bushenjiannao 300 mL bid (39)	Piracetam 800 mg tid + hydergine 2 tablet bid (34)	MMSE, HDS, hemodynamic changes, “protocol for new herbal drugs study on dementia”^∗b^	N	N	U
41	Chen et al. [84], China Monotherapy Single center	Randomized: method not mentioned; double blind: method not mentioned; parallel design; 3 months duration	68 VaD patients; age: 60–86; duration: 1–7 yr	DSM-4, HDS, MMSE, CT, MRI	Shenlong 600 mL/day (36)	Piracetam 1.6 g tid (32)	MMSE, BBS, HDS, “protocol for new herbal drugs study on dementia”^∗b^	N	N	U
42	Cui et al. [77], China Monotherapy Single center	Randomized: computer generated sequence; double blinding not mentioned; parallel design; 3 months duration	67 VaD patients; age: 56–83; duration: 2 m–31 m	DSM-4, NINDS-AIREN, HIS, CDR	Shuangshencuzhi 8 g tid (51)	Duxil 1 tablet bid (16)	MMSE, BBS, HCY, CT, MRI	N	5	U
43	He [57], China Monotherapy Single center	Randomized: method not mentioned; single blind: method not mentioned; parallel design; 8 weeks duration	90 VaD patients; age: 48–80; duration: 2 m–6.5 yr	NINDS-AIREN, SDSVD, CDR, HIS, “criteria for the diagnosis, the differentiation of syndrome and the evaluation of efficacy of vascular dementia for research studies”^∗ g^	Kangnao 6 g tid (60)	Hydergine 2 mg tid (30)	MMSE, hemodynamic changes, SELF	N	N	N
44	Jin et al. [85], China Monotherapy Unclear center	Randomized: method not mentioned; double blind: details given; crossover design (12 w + 4 w wash out + 12 w);	72 VaD patients; age: 55–83; duration: 1–7 yr	DSM-4-R, MMSE, ADL, HDS, CT, MRI, “protocol for new herbal drugs study on dementia”^∗ b^	Jiannaoyizhi 2 g tid (72)	Placebo tid (72)	MMSE-R, HDS, ADL-R, “protocol for new herbal drugs study on dementia”^∗b^, “protocol for new herbal drugs study on stroke”^∗ b^	N	N	Y (2, sore throat, mouth dryness)
45	Yan [58], China Adjunct therapy Single center	Randomized: method not mentioned; double blinding not mentioned; parallel design; 1 month duration	79 VaD patients; age: 51–78; duration: 0.5–9 yr.	DSM-4-R, HDS-R, MMSE, CT, MRI	Buyanghuanwu + Cerebroprotein Hydrolysate 10 mL 20 d, Aniracetam 0.12 g tid; Nimodipine 20 mg tid (36)	Cerebroprot-ein Hydrolysate 10 mL 20 d, Aniracetam 0.12 g tid; Nimodipine 20 mg tid (43)	HDS-R	N	N	U
46	Chang et al. [70], China Monotherapy Single center	Randomized: method not mentioned; double blinding not mentioned; parallel design; 12 weeks duration	66 VaD patients; age: 60–78; duration: 1.3–3.6 yr	DSM-4, HIS, “TCM diagnostic criteria, differentiation and outcome measures on senile dementia,”^∗c^ MMSE, CT, MRI, HDS	Qihong 300 mL bid (33)	Piracetam 0.4 g bid (33)	HDS, MMSE	N	N	U
47	Li et al. [103], China Monotherapy Single center	Randomized: method not mentioned; double blinding not mentioned; parallel design; 2 months duration	120 VaD patients; age: 59–75; duration: 0.6–6.2 yr	DSM-4, MMSE-R, CDR, NINDS-AIREN, SDSVD, HIS, CSDD, “protocol for new herbal drugs study on dementia”^∗ b^	Shouwuyizhi 2.4 g tid (80)	Piracetam 0.8 g tid (40)	MMSE, HDS, WMS, SDSVD, TCD, hemodynamic changes, SELF	N	N	N

Key: ^∗1^: N: intention-to-treat analysis is not used; Y: intention-to-treat analysis is applied.

^∗2^: N: no report of drop-out; Y: drop-out reported (with no. of dropouts in bracket).

^∗3^: N: report as no adverse events; Y: adverse events reported (with no. and details in bracket); U= adverse events unknown.

^∗a^: Quan et al. [104], ^∗b^: Guidance principle of clinical study on new drug of traditional Chinese medicine [105], ^∗c^: Fu [106], ^∗d^: Zhou [107], ^∗e^: Huang and Liu [108], ^∗f^: Wang et al. [109], ^∗f^: Tian et al. [88].

BEAM: brain electrical activity mapping

**Table 4:** Statistics on herbal intervention.

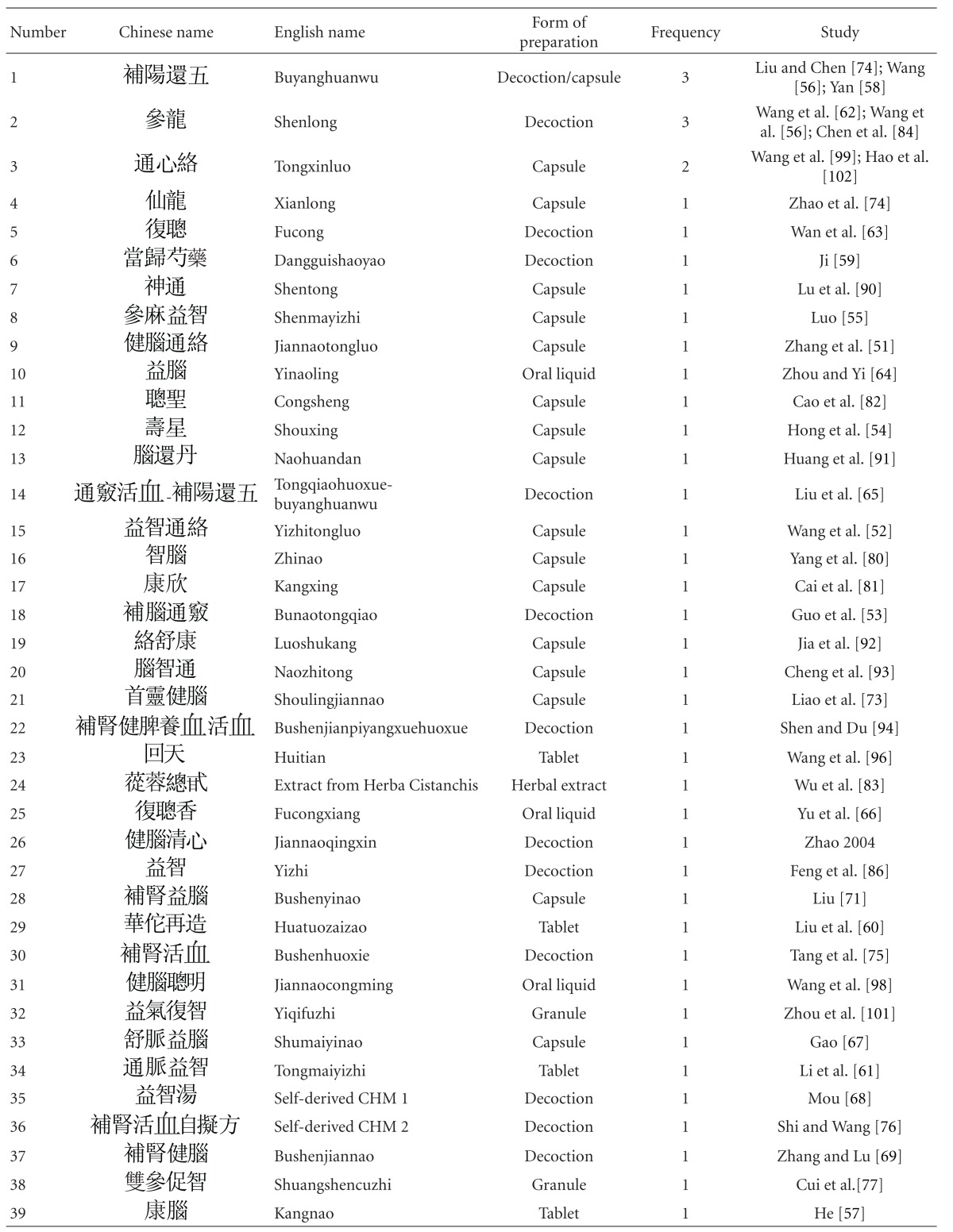
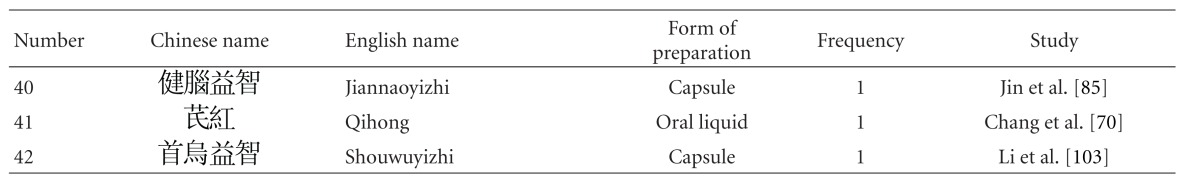

**Table 5:** The 30 most commonly used herbal constituents.

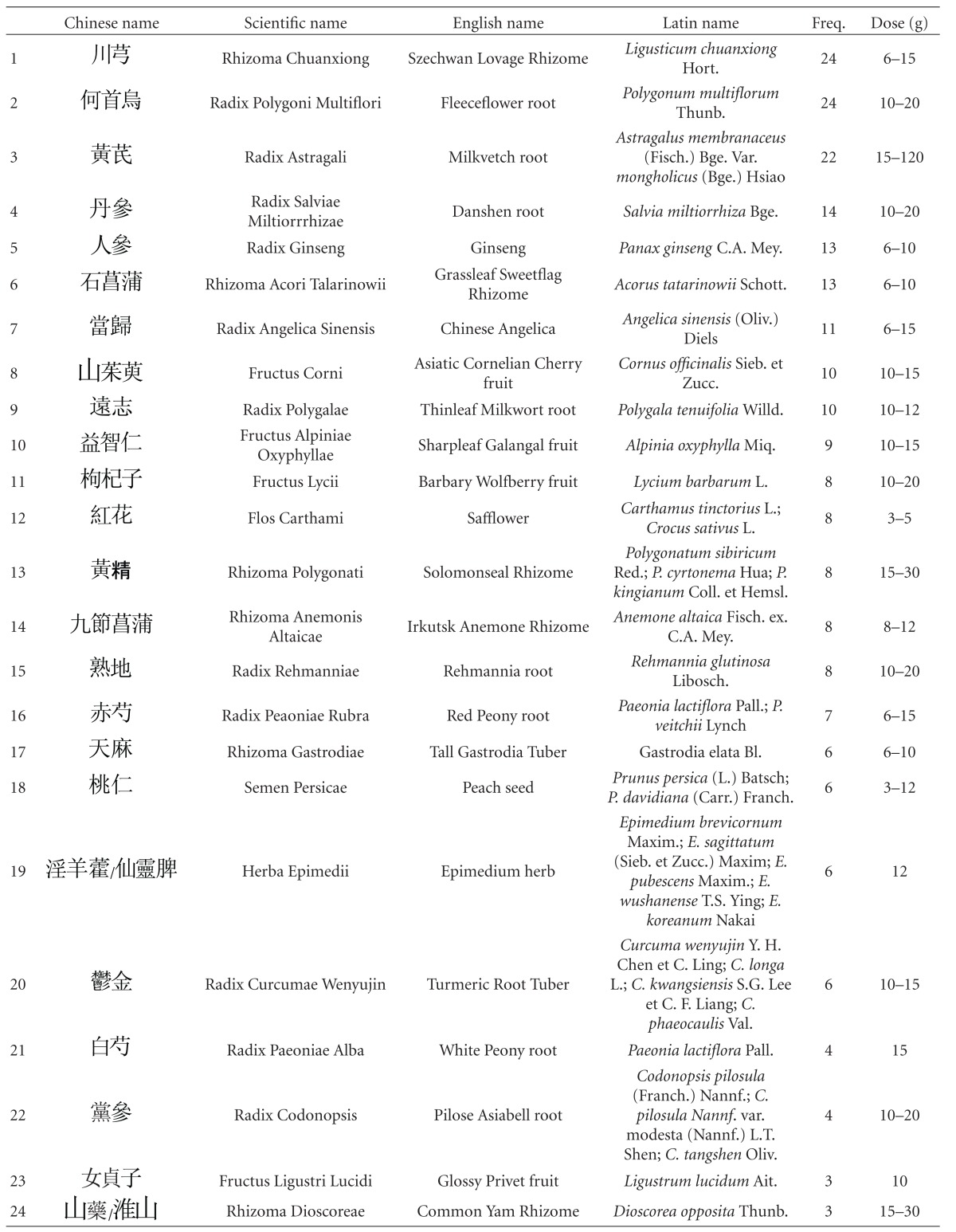
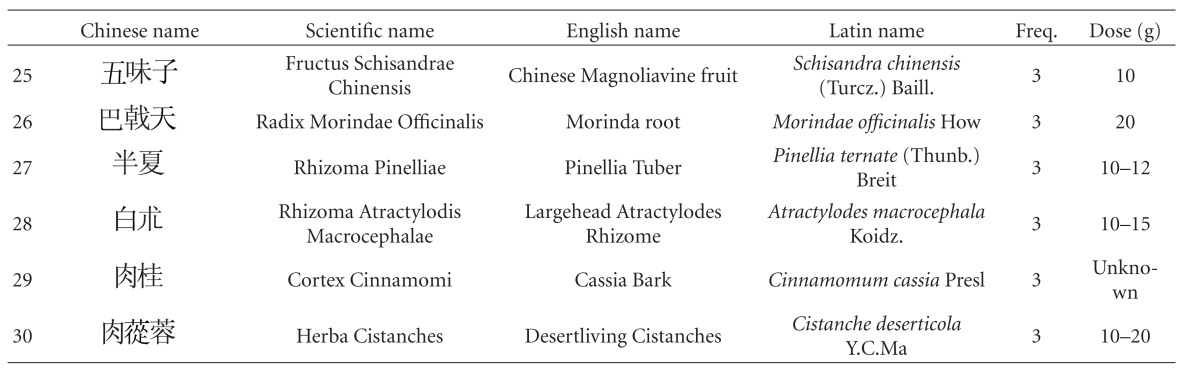

## References

[1] Quinn J (2003). Vascular dementia. *Journal of the American Medical Directors Association*.

[2] de Leeuw FE, van Gijn J (2003). Vascular dementia. *Practical Neurology*.

[3] Jellinger KA (2008). The pathology of “vascular dementia”: a critical update. *Journal of Alzheimers Disease*.

[4] Román GC (2003). Vascular dementia: distinguishing characteristics, treatment, and prevention. *Journal of the American Geriatrics Society*.

[5] Hentschel F, Supprian T, Frölich L (2005). Alzheimer’s disease versus vascular dementia—dichotomy or interaction?. *Fortschritte der Neurologie Psychiatrie*.

[6] Schneck MJ (2008). Vascular dementia. *Topics in Stroke Rehabilitation*.

[7] Moretti R, Torre P, Antonello RM, Cazzato G (2006). Behavioral alterations and vascular dementia. *Neurologist*.

[8] Swanson KA, Carnahan RM (2007). Dementia and comorbidities: an overview of diagnosis and management. *Journal of Pharmacy Practice*.

[9] Schneider B, Maurer K, Frölich L (2001). Dementia and suicide. *Fortschritte der Neurologie Psychiatrie*.

[10] Kalaria RN, Maestre GE, Arizaga R (2008). Alzheimer’s disease and vascular dementia in developing countries: prevalence, management, and risk factors. *The Lancet Neurology*.

[11] Kalaria R (2002). Similarities between Alzheimer’s disease and vascular dementia. *Journal of the Neurological Sciences*.

[12] McVeigh C, Passmore P (2006). Vascular dementia: prevention and treatment. *Clinical Interventions in Aging*.

[13] Dong MJ, Peng B, Lin XT, Zhao J, Zhou YR, Wang RH (2007). The prevalence of dementia in the People’s Republic of China: a systematic analysis of 1980–2004 studies. *Age and Ageing*.

[14] Andersen CK, Søgaard J, Hansen E (1999). The cost of dementia in Denmark: the Odense study. *Dementia and Geriatric Cognitive Disorders*.

[15] Hill J, Fillit H, Shah SN, del Valle MC, Futterman R (2005). Patterns of healthcare utilization and costs for vascular dementia in a community-dwelling population. *Journal of Alzheimer’s Disease*.

[16] Moretti R, Torre P, Antonello RM, Vilotti C, Pizzolato G (2007). New treatment options for vascular dementia. *Aging Health*.

[17] Zekry D (2009). Is it possible to treat vascular dementia?. *Frontiers of Neurology and Neuroscience*.

[18] Baskys A, Hou AC (2007). Vascular dementia: pharmacological treatment approaches and perspectives. *Clinical Interventions in Aging*.

[19] Pantoni L (2004). Treatment of vascular dementia: evidence from trials with non-cholinergic drugs. *Journal of the Neurological Sciences*.

[20] Sorrentino G, Migliaccio R, Bonavita V (2008). Treatment of vascular dementia: the route of prevention. *European Neurology*.

[21] Stephan BC, Brayne C (2008). Vascular factors and prevention of dementia. *International Review of Psychiatry*.

[22] Perry N, Court G, Bidet N, Court J, Perry E (1996). European herbs with cholinergic activities: potential in dementia therapy. *International Journal of Geriatric Psychiatry*.

[23] Yarnell E (1998). Lemonbalm: humble but potent herb. *Alternative and Complementary Therapies*.

[24] Manyam BV (1999). Dementia in Ayurveda. *Journal of Alternative and Complementary Medicine*.

[25] Upton R (200). *Ashwagandha Root. Withania Somnifera. Analytical, Quality Control and Therapeutic Monograph*.

[26] Howes MJ, Perry NS, Houghton PJ (2003). Plants with traditional uses and activities, relevant to the management of Alzheimer’s disease and other cognitive disorders. *Phytotherapy Research*.

[27] Chang H, But PP (1987). *Pharmacology and Applications of Chinese Materia Medica*.

[28] Kenner D, Requena Y (1996). *Botanical Medicine: A European Professional Perspective*.

[29] Boughon F (1998). Herbs for the elderly. *Australian Journal of Medical Herbalism*.

[30] Zbinden S, Seiler C (2002). Phytotherapy in cardiovascular medicine. *Therapeutische Umschau*.

[31] Klusa V, Germane S, Nöldner M, Chatterjee SS (2001). Hypericum extract and hyperforin: memory-enhancing properties in rodents. *Pharmacopsychiatry*.

[32] Tam WY, Chook P, Qiao M (2009). The efficacy and tolerability of adjunctive alternative herbal medicine (Salvia miltiorrhiza and Pueraria lobata) on vascular function and structure in coronary patients. *Journal of Alternative and Complementary Medicine*.

[33] Cai J, Zhou F, Du J (2008). Effects of glossy privet fruit on neural cell apoptosis in the cortical parietal lobe and hippocampal CA1 region of vascular dementia rats. *Neural Regeneration Research*.

[34] Cho K, Noh K, Jung W (2008). A preliminary study on the inhibitory effect of Chunghyul-dan on stroke recurrence in patients with small vessel disease. *Neurological Research*.

[35] Wang R, Yan H, Tang XC (2006). Progress in studies of huperzine A, a natural cholinesterase inhibitor from Chinese herbal medicine. *Acta Pharmacologica Sinica*.

[36] Man SC, Durairajan SS, Kum WF (2008). Systematic review on the efficacy and safety of herbal medicines for Alzheimer’s disease. *Journal of Alzheimer’s Disease*.

[37] Viswanathan A, Rocca WA, Tzourio C (2009). Vascular risk factors and dementia: how to move forward?. *Neurology*.

[38] Murray ME, Knopman DS, Dickson DW (2007). Vascular dementia: clinical, neuroradiologic and neuropathologic aspects. *Panminerva Medica*.

[39] American Psychiatric Association (1987). *Diagnostic and Statistical Manual of Mental Disorders: DSM-III-R*.

[40] American Psychiatric Association (1994). *Diagnostic and Statistical Manual of Mental Disorders: DSM-IV*.

[41] World Health Organization (1992). *The ICD-10 Classification of Mental and Behavioural Disorders : Clinical Descriptions and Diagnostic Guidelines*.

[42] Chui HC, Victoroff JI, Margolin D, Jagust W, Shankle R, Katzman R (1992). Criteria for the diagnosis of ischemic vascular dementia proposed by the State of California Alzheimer’s Disease Diagnostic and Treatment Centers. *Neurology*.

[43] Hachinski VC, Iliff LD, Zilhka E (1975). Cerebral blood flow in dementia. *Archives of Neurology*.

[44] Roman GC, Tatemichi TK, Erkinjuntti T (1993). Vascular dementia: diagnostic criteria for research studies: report of the NINDS-AIREN International Workshop. *Neurology*.

[45] Folstein MF, Folstein SE, McHugh PR (1975). ’Mini mental state’. A practical method for grading the cognitive state of patients for the clinician. *Journal of Psychiatric Research*.

[46] Katz S (2006). Activities of daily living scale. *Home Health Care Services Quarterly*.

[47] Tsai N, Gao ZX (1989). Validity of Hasegawa’s dementia scale for screening dementia among aged Chinese. *International Psychogeriatrics*.

[48] Zhang W, Leonard T, Bath-Hextall F (2007). Chinese herbal medicine for atopic eczema. *Cochrane Database of Systematic Reviews*.

[49] Moher D, Schulz KF, Altman DG (2001). The CONSORT statement: revised recommendations for improving the quality of reports of parallel-group randomised trials. *Lancet*.

[50] Moher D, Schulz KF, Altman DG (2003). The CONSORT statement: revised recommendations for improving the quality of reports of parallel-group randomised trials. *Clinical Oral Investigations*.

[51] Zhang CQ, Jin YL, Zhao LJ (2001). Clinical study on Jian Nao Tong Luo Jiao Nang in the treatment of vascular dementia. *Chinese Journal of Information on Traditional Chinese Medicine*.

[52] Wang FJ, Lu SJ, Dong Z (2002). Clinical study on Yi Zhi Tong Luo Jiao Nang in the treatment of vascular dementia. *Modern Journal of Integrated Chinese Traditional and Western Medicine*.

[53] Guo XF, Yan YB, Zhao YH, Sun CX (2003). Clinical study on Bu Nao Tong Qiao Fang in the treatment of vascular dementia. *Journal of Traditional Chinese Medicine*.

[54] Hong ML, Hou GD, Hong LS (2002). Clinical study on Shou Xing Jiao Nang in the treatment of vascular dementia. *Hubei Journal of Traditional Chinese Medicine*.

[55] Luo ZG, Zhou WQ, Gao P, Cui L (2001). Clinical study on treatment of senile vascular dementia with Shen Ma Yi Zhi capsule. *Journal of Traditional Chinese Medicine*.

[56] Wang FW, Wang ZK, Jiang N (2005). Clinical study on the effect of shenlong decoction on vascular dementia. *Academic Journal of PLA Postgraduate Medical School*.

[57] He H (2007). Clinical study on Yi Shen Hua Zhuo Qu Yu Zhu Tan Fa in the treatment of vascular dementia. *Chinese Journal of Information on Traditional Chinese Medicine*.

[58] Yan Q (2007). Clinical study on Chinese medicine in the treatment of vascular dementia. *Journal of Sichuan of Traditional Medicine*.

[59] Ji H (2000). Clinical observation on the treatment of vaseular dementia by Danggui Shaoyao powder. *Shanxi Journal of Traditional Chinese Medicine*.

[60] Liu HJ, Qin J, Liang W, Huang YQ (2005). Clinical study on Hua Tuo Zai Zao Wan in the treatment of vascular dementia. *Chinese Traditional and Herbal Drugs*.

[61] Li LJ, Zhang LT, Li YM, Shang DS (2006). Clinical study on Tong Mai Yi Zhi Wan in the treatment of vascular dementia. *Journal of Hubei College of Traditional Chinese Medicine*.

[62] Wang FW, Jiang N, Tong ZQ (2004). Clinical study on shenlong tang for improving neurological impairment in patients with vascular dementia. *Chinese Journal of Clinical Rehabilitation*.

[63] Wan Y, Wang YF (1998). Clinical study on effect of fucongtang in treating vascular dementia. *Hebei Medicine*.

[64] Zhou GP, Yi X (2001). Clinical study on Yi Nao Ling in the treatment of vascular dementia. *Shandong Journal of Traditional Chinese Medicine*.

[65] Liu CZ, Zhou LH, Shui Z (2002). Clinical study on Tong Qiao Huo Xue Tang He Bu Yang Huan Wu Tang in the treatment of vascular dementia. *Journal of Traditional Chinese Medicine*.

[66] Yu SZ, Zheng GS, Shi Q, Ai LX, Cheng RR (2004). Clinical study on Fucong Xiangye for treatment of 72 cases of vascular dementia. *Journal of Traditional Chinese Medicine*.

[67] Gao Q (2006). Clinical study on Shu Mai Yi Nao Jiao Nang in the treatment of vascular dementia. *Forum on Traditional Chinese Medicine*.

[68] Mou WH (2006). Clinical observation of Yizhi decoction treating senile vascular dementia. *Liaoning Journal of Traditional Chinese Medicine*.

[69] Zhang WY, Lu ZP (2006). Clinical study on effect of Bushenjiannao decoction in vascular dementia patients. *Chinese Journal of Integrative Medicine on Cardio-/Cerebrovascular Disease*.

[70] Chang ZY, Qiao QC, Zhang YH, Qiao QZ (2008). Clinical study on Qi Hong Kou Fu Ye in the treatment of vascular dementia. *Chinese Remedies & Clinics*.

[71] Liu GT (2005). Changes of intellectual status, hemorheology and blood lipids in patients with vascular dementia and the interventional effects of Bushen Yinao capsule. *Chinese Journal of Clinical Rehabilitation*.

[72] Cai J, Du J, Huang JS, Lin QC (2003). Clinical effect of the principle of tonifying-kidney, invigorating-spleen, nourishing and activating-blood on patients with vascular dementia. *Chinese Journal of Clinical Rehabilitation*.

[73] Liao XL, Chen KY, Zheng ZR, Liu H, Zheng KY (2004). Clinical study of Shoulingjiannao capsule in improvement of brain function for patients with vascular dementia. *Chinese Journal of Clinical Rehabilitation*.

[74] Liu W, Chen ZY (2005). Clinical study of shulzhltong capsule in treating senile vascular dementia. *China and Foreign and Medical Journal*.

[75] Tang XJ, Lao YR, Yang ZM, Huang PX, Luo XD (2005). Clinical observation on modified bushen huoxie decoction for the treatment of vascular dementia. *Journal of Guangzhou University of Traditional Chinese Medicine*.

[76] Shi H, Wang HM (2006). Clinical effects observation of invigorating kidney and promoting blood flow method on treating vascular dementia. *Tianjin Journal of Traditional Chin Medicine*.

[77] Cui L, Liu F, Guo MD (2007). Clinical study on Shuang Shen Cu Zhi Ke Li in the treatment of vascular dementia. *China Journal of Experimental Traditional Medical Formulae*.

[78] Li YP, Shi ZD (1999). Clinical study on Bu Yang Huan Wu Tang in the treatment of vascular dementia. *Journal of Hubei College of Traditional Chinese Medicine*.

[79] Zhao Y, Zhou W, Gao P (1999). Clinical study on effect of xianlong capsule on senile vascular dementia. *Chinese Journal of Integrated Traditional and Western Medicine*.

[80] Yang WM, Lu WJ, Han MX (2002). Clinical research of Zhinao capsule on 60 cases with vascular dementia. *China Journal of Experimental Traditional Medical Formulae*.

[81] Cai J, Du J, Huang JS, Lin QC, Chen KJ (2003). Clinical observation of Kangxin capsule on the influence of vascular dementia. *Chinese Journal of Gerontology*.

[82] Cao XL, Song XX, Hu ZQ, Mei T, Zhou J, Zhang FR (2002). Clinical study on treatment of vascular dementia with congsheng capsule. *Journal of Emergency in Traditional Chinese Medicine*.

[83] Wu SY, Fang SQ, Liang H (2004). Clinical study on Cong Rong Zong Dai Jiao Nang in the treatment of vascular dementia. *Fujian Journal of Traditional Chinese Medicine*.

[84] Chen LP, Wang FW, Jia JJ, Hao AJ (2007). Effects of shenlong decoction on life quality of the aged patients with cerebral vascular dementia. *Medical Journal of Chinese People’s Liberation Army*.

[85] Jin XM, Li L, Lian LX, Song ZH (2007). Clinical investigation of Jiannaoyizhi capsule to improving vascular dementia sufferer on nerve function damage. *Chinese Archives of Traditional Chinese Medicine*.

[86] Feng YY, Zhang JX, Ding XD (2005). Clinical study on Yi Zhi Tang in the treatment of vascular dementia. *Study Journal of Traditional Chinese Medicine*.

[87] Morris JC (1993). The clinical dementia rating (CDR): current version and scoring rules. *Neurology*.

[88] Tian JZ, Ming XH, Jin WT (2002). Criteria for the diagnosis, the differentiation of syndrome and the evaluation of efficacy of vascular dementia for research studies. *Chinese Journal of Gerontology*.

[89] Fillenbaum GG, Smyer MA (1981). The development, validity, and reliability of the OARS multidimensional functional assessment questionnaire. *Journals of Gerontology*.

[90] Lu H, Wang YY, Chen F, Wu Y, Gai DH, Cui Z (2000). Clinical observation on 50 cases of cerebrovascular dementia treated with Shentong capsule. *J TCM*.

[91] Huang QH, Li QM, Tan ZH (2002). Clinical study on naohuandan capsule in the treatment of senile dementia. *Practical Geriatrics*.

[92] Jia WH, Ding XN, Xu ZM, Jie MP (2003). Clinical study on the Luoshukang Capsule in the treatment of vascular dementia [Luoshukang Jiao Nang Zhi Liao Xue Guan Xing Chi Dai De Lin Chuang Yan Jiu]. *Guang Ming Zhong Yi*.

[93] Cheng WP, Ma L, Toshihide H, Wang QW (2004). Treatment of Vascular Dementia by Naozhitong Capsule: A Clinical Observation of 18 Cases. *J New CM*.

[94] Shen SH, Du J (2004). The therapeutic effects of Kidney-Tonifying and spleen-invigorating, blood-nourishing and quickening the blood formula on vascular dementia in the pattern of Kidney deficiency and blood stasis. *J TCM U HUNAN*.

[95] Wang FW, Jiang N, Tong ZQ (2004). Clinical study on Shenlong Tang for improving neurological impairment in patients with vascular dementia. *CHIN J CLIN REHAB*.

[96] Wang J, Chen QX, Hai Y (2004). Clinical Study of Huitianpian in the treatment of vascular dementia. *Chinese Archieves of Traditional Chinese Medicine*.

[97] Zhao YJ (2004). A Summary on 50 Cases of Vascular Dementia Treated by Jiannao Qingxin Decoction. *HUNAN J TRAD CHIN MED*.

[98] Wang J, Chen QW, Bai L (2005). Clinical study of Jiannaocongming decoction in the treatment of vascular dementia [Jiannaocongming Kou Fu Ye Zhi Liao Xue Guan Xing Chi Dai De Lin Chuang Yan Jiu]. *LIAONING J TCM*.

[99] Wang LL, Ying PZ, Wang XM (2005). Clinical efficacy of Tongxinluo capsule in the treatment of vascular dementia. *CHIN J DIFFIC COMPL CAS*.

[100] Wang ZY (2005). Clinical observation of Buyanghuanwutang in the treatment of vascular dementia [Buyanghuanwutang jia xi yao zhi liao xue guan xing chi dai liao xiao guan cha]. *J SICHUAN TRAD CHIN MED*.

[101] Zhou JY, Liu T, Sun CC (2005). Clinical Study on “Yiqi Fuzhi Granule” in Treating Vascular Dementia. *SHANGHAI J TRAD CHIN MED*.

[102] Hao WP, Ye FH, Li LZ (2006). Clinical observations of Tongxingluo capsule in the treatment of vascular dementia. *J CLIN PSYCHOSOM DIS*.

[103] Li CS, Li J, Guan XH (2008). Clinical study of Shouwuyizhi capsule in the treatment of vascular dementia. *CHIN J GERI*.

[104] Quan ZQ (1996). Progress on the TCM diagnosis and treatment of vascular dementia. *Acta Chinese Medicine and Pharmacology*.

[105] *Guidance Principle of Clinical Study on New Drug of Traditional Chinese Medicine*.

[106] Fu RJ (1991). TCM diagnostic criteria, differentiation and outcome measures on senile dementia. *Journal of Traditional Chinese Medicine*.

[107] Zhou WQ (1986). Protocol for the selection of anti-aging herbal medicine and the corresponding outcome measures. *Chinese Journal of Integrated and Traditional and Western Medicine*.

[108] Huang PX, Liu MC (2000). *TCM manual for neurological diseases*.

[109] Wang XD (1997). *Diagnostic manual for geriatrics*.

[110] Dharmananda S (1996). Alzheimer’s disease: treatment with Chinese herbs.

[111] Xiong L, Fang ZY, Tao XN, Bai M, Feng G (2007). Effect and mechanism of ligustrazine on Th1/Th2 cytokines in a rat asthma model. *American Journal of Chinese Medicine*.

[112] Cai D (2008). Effectiveness and safety of xiaoshuanchangrong (XSCR) capsule for the treatment of patients who have suffered from a stroke. *Fudan University*.

[113] Ling S, Nheu L, Dai A, Guo Z, Komesaroff P (2008). Effects of four medicinal herbs on human vascular endothelial cells in culture. *International Journal of Cardiology*.

[114] Chan YC, Wang MF, Chen YC, Yang DY, Lee MS, Cheng FC (2003). Long-term administration of Polygonum multiflorum Thunb. reduces cerebral ischemia-induced infarct volume in gerbils. *American Journal of Chinese Medicine*.

[115] Ip SP, Tse AS, Poon MK, Ko KM, Ma CY (1997). Antioxidant activities of Polygonum multiflorum Thunb; in vivo and in vitro. *Phytotherapy Research*.

[116] Um MY, Choi WH, Aan JY, Kim SR, Ha TY (2006). Protective effect of Polygonum multiflorum Thunb on amyloid *β*-peptide 25-35 induced cognitive deficits in mice. *Journal of Ethnopharmacology*.

[117] Chan YC, Cheng FC, Wang MF (2002). Beneficial effects of different Polygonum multiflorum Thunb. Extracts on memory and hippocampus morphology. *Journal of Nutritional Science and Vitaminology*.

[118] Chiu MJ (2005). Efficacy and safety study of DCB-AD1 in patients with mild to moderate Alzheimer’s disease. *National Taiwan University Hospital*.

[119] Zhang ZJ (2004). Therapeutic effects of herbal extracts and constituents in animal models of psychiatric disorders. *Life Sciences*.

[120] Zhang Q, Chang Z, Yang J, Wang Q (2008). Antiatherogenic property of triterpenoids-enriched extract from the aerial parts of Salvia miltiorrhiza. *Phytotherapy Research*.

[121] Lao CJ, Lin JG, Kuo JS (2003). Effect of Salvia Miltiorrhiza Bunge on cerebral infarct in ischemia-reperfusion injured rats. *American Journal of Chinese Medicine*.

[122] Zhang G, Liu A, Zhou Y, San X, Jin T, Jin Y (2007). Panax ginseng ginsenoside-Rg2 protects memory impairment via anti-apoptosis in a rat model with vascular dementia. *Journal of Ethnopharmacology*.

[123] Huang YC, Chen CT, Chen SC (2005). A natural compound (Ginsenoside Re) isolated from Panax ginseng as a novel angiogenic agent for tissue regeneration. *Pharmaceutical Research*.

[124] Lee ST, Chu K, Sim JY, Heo JH, Kim M (2008). Panax ginseng enhances cognitive performance in Alzheimer disease. *Alzheimer Disease and Associated Disorders*.

[125] Liao WP, Chen L, Yi YH (2005). Study of antiepileptic effect of extracts from Acorus tatarinowii schott. *Epilepsia*.

[126] Wang W, Sun F, An Y (2009). Morroniside protects human neuroblastoma SH-SY5Y cells against hydrogen peroxide-induced cytotoxicity. *European Journal of Pharmacology*.

[127] Li C, Yang J, Yu S (2008). Triterpenoid saponins with neuroprotective effects from the roots of Polygala tenuifolia. *Planta Medica*.

[128] Naito R, Tohda C (2006). Characterization of anti-neurodegenerative effects of polygala tenuifolia in abeta(25-35)-treated cortical neurons. *Biological and Pharmaceutical Bulletin*.

[129] Koo BS, Lee WC, Chang YC, Kim CH (2004). Protective effects of alpinae Oxyphyllae fructus (Alpinia oxyphylla MIQ) water-extracts on neurons from ischemic damage and neuronal cell toxicity. *Phytotherapy Research*.

[130] Yu X, An L, Wang Y, Zhao H, Gao C (2003). Neuroprotective effect of Alpinia oxyphylla Miq. Fruits against glutamate-induced apoptosis in cortical neurons. *Toxicology Letters*.

[131] Zhang F, Zhang J, Wang L, Mao D (2008). Effects of polygonatum sibiricum polysaccharide on learning and memory in a scopolamine-induced mouse model of dementia. *Neural Regeneration Research*.

[132] Lee SM, Yoon MY, Park HR (2008). Protective effects of Paeonia lactiflora pall on hydrogen peroxide-induced apoptosis in PC12 cells. *Bioscience, Biotechnology and Biochemistry*.

[133] Zhang TX, Niu CQ, Jing HE, Liu H (2008). Effect of water decoction of the Gastrodia elata BI on vasodilation of rabbit aorta in vitro. *Journal of Clinical Rehabilitative Tissue Engineering Research*.

[134] Ahn EK, Jeon HJ, Lim EJ, Jung HJ, Park EH (2007). Anti-inflammatory and anti-angiogenic activities of Gastrodia elata Blume. *Journal of Ethnopharmacology*.

[135] Liu JH, Bao YM, Song JJ, An LJ (2003). Codonopsis pilosula (Franch) Nannf total alkaloids potentiate neurite outgrowth induced by nerve growth factor in PC12 cells. *Acta Pharmacologica Sinica*.

[136] Julian PTH, Sally G, Jonathan JD, Julian PTH, Douglas GA (2008). Summarizing effects across studies. *Cochrane Handbook for Systematic Reviews of Interventions*.

